# Molecular Characterization of Publicly Available NDV F Gene Sequences From Bangladesh (2010–2024)

**DOI:** 10.1155/vmi/8821675

**Published:** 2026-08-24

**Authors:** Farah Zereen, Md. Abdur Rahman, Md. Golzar Hossain, Jahangir Alam, Masaru Shimada, Md. Tanvir Rahman, Muhammad Munir, Sukumar Saha

**Affiliations:** ^1^ Department of Microbiology and Hygiene, Bangladesh Agricultural University, Mymensingh 2202, Bangladesh, bau.edu.bd; ^2^ Department of Microbiology, Gono Bishwabidyalay, Dhaka, Bangladesh; ^3^ Department of Animal Production, Gono Bishwabidyalay, Dhaka, Bangladesh; ^4^ Animal Biotechnology Division, National Institute of Biotechnology, Dhaka, Bangladesh, nib.gov.bd; ^5^ Department of Molecular Biodefense Research, Graduate School of Medicine, Yokohama City University, Yokohama 236-0004, Japan, yokohama-cu.ac.jp; ^6^ Division of Biomedical and Life Sciences, Faculty of Health and Medicine, Lancaster University, Lancaster, UK, lancs.ac.uk

**Keywords:** Bangladesh, fusion gene, genotyping, Newcastle disease virus, phylogenetics, vaccine mismatch

## Abstract

Newcastle disease, caused by avian orthoavulavirus serotype 1, is a highly contagious and economically devastating viral infection affecting both wild and domestic birds. The disease is characterized by severe respiratory, gastrointestinal, and neurological symptoms, often leading to high mortality and substantial production losses, particularly in commercial poultry. In Bangladesh, multiple virulent and avirulent Newcastle disease virus (NDV) genotypes and subgenotypes continue to circulate despite ongoing vaccination programs. This research presents a comprehensive analysis of the genetic landscape of prevalent NDV strains in Bangladesh between 2010 and 2024 using full‐length coding sequences of the fusion (F) gene. Specifically, we focused on genotypes, subgenotypes, host species, and annual distribution patterns in the country. We further investigated phylogenetic relationships, evolutionary divergence, and amino acid substitutions in the full‐length F protein. Comparative analyses of amino acid variability and sequence similarity between currently used vaccine strains and prevalent Bangladeshi field isolates were also scrutinized. We have unraveled considerable genetic divergence, and highlighted mismatches in genotypic coverage. These findings underscore the urgent need to reevaluate existing NDV vaccines strain to ensure improved protection against circulating NDVs in Bangladesh.

## 1. Introduction

Newcastle disease (ND) is a highly fatal and economically important viral infection of birds, particularly poultry, caused by Newcastle disease virus (NDV), a prototype member of the orthoavulavirus serotype 1 [1]. The NDV infection is not only limited to commercial poultry but can also infect a wide range of wild bird species [2]. It was first reported in 1926 in Indonesia and then traveled to England and Sri Lanka [3, 4], triggering a global panzootic [5], while Bangladesh first reported ND outbreaks in 1981 [6]. Clinical manifestations include neurological signs, gastrointestinal disorders, respiratory illness, and reduced egg production, while severe cases often lead to sudden death [7].

NDV, also known as avian orthoavulavirus 1 (AOAV‐1), belongs to the genus *Orthoavulavirus*, subfamily *Avulavirinae*, family *Paramyxoviridae*, and order *Mononegavirales*, according to the International Committee on Taxonomy of Viruses (ICTV) classification [8]. It is a nonsegmented, negative‐sense, single‐stranded RNA virus with a genome of approximately 15,000 nucleotides that encodes six structural proteins: hemagglutinin–neuraminidase (HN), RNA‐dependent RNA polymerase (L), matrix (M), fusion (F), phosphoprotein (P), and nucleocapsid protein (NP) [9, 10]. The NP encapsidates the viral RNA genome, forming a ribonucleoprotein (RNP) complex that protects the genome from degradation and serves as a template for replication and transcription. The P acts as an essential cofactor of the viral RNA‐dependent RNA polymerase, stabilizing the L protein and facilitating RNA synthesis. The L protein functions as the catalytic subunit of the polymerase complex, mediating viral transcription and genome replication. The M protein lines the inner surface of the viral envelope and plays a critical role in virion assembly, budding, and modulation of host cellular processes. The two surface glycoproteins, F and HN, are key determinants of viral infectivity and virulence: The HN protein mediates viral attachment to host cell receptors via binding to sialic acid and exhibits neuraminidase activity, while the F protein facilitates fusion of the viral envelope with the host cell membrane after proteolytic activation, enabling viral entry and cell‐to‐cell spread. Collectively, these proteins coordinate viral entry, replication, assembly, and release, as well as interactions with host immune responses [11–13].

The NDV genome exhibits marked genetic diversity, with strains varying in pathogenicity [14]. The NDV is classified into three pathotypes based on virulence: velogenic, mesogenic, and lentogenic [15]. Velogenic strains cause severe systemic disease and high mortality [16]; mesogenic strains primarily affect the respiratory and nervous systems with moderate mortality [17, 18]; and lentogenic strains generally cause mild or asymptomatic infections, particularly in the intestines [19].

Phylogenetic analysis based on F and HN genes provide high‐resolution clustering patterns and complement whole‐genome analyses. However, the complete F gene sequence is the preferred target for comprehensive NDV studies, including virulence evaluation, pathotyping, genotyping, and phylogenetic analysis, as recommended by World Organization for Animal Health (WOAH) [15, 20]. Based on full‐length F gene sequences, NDV is divided into two major classes: Class I and Class II [21]. Class I strains are generally avirulent and mostly detected in wild aquatic birds and live bird markets [22, 23]. In contrast, Class II strains are typically virulent and are frequently recovered from domestic poultry as well as wild birds [24, 25]. Class I consists of a single genotype with three subgenotypes, whereas Class II encompasses at least 21 genotypes (I–XXI), each containing several subgenotypes [26]. NDV has a long history in Southeast Asia, where genotypes II, IV, VII, and XIII continue to circulate simultaneously [27]. Preventive vaccination is the main strategy for controlling ND in commercial poultry, yet outbreaks continue to occur in vaccinated flocks due to circulating virulent strains of NDV, causing losses from death, poor growth, and reduced egg production.

A key determinant of NDV virulence is the amino acid motif at the F protein cleavage site (residues 112–117) [28]. Lentogenic strains typically harbor a monobasic motif (112G‐R/K‐Q‐G‐R↓L117), whereas mesogenic and velogenic strains carry a multibasic motif (112R/G/K‐R‐Q/K‐K/R‐R↓F117), which enables efficient cleavage and viral entry [29]. Characterizing the F protein cleavage site is critical for assessing virulence, guiding vaccine design, and understanding NDV evolution [30]. The high genetic variability of NDV in poultry populations highlights the need for continuous molecular surveillance [26] using the complete F or HN gene, or ideally, whole‐genome sequences.

Therefore, this research investigates the genetic diversity of NDV isolates in Bangladesh from 2010 to 2024 using full‐length coding sequences of the F gene available in the NCBI GenBank database, focusing on phylogenetic classification, evolutionary divergence, and genotype mismatches between vaccine strains and circulating viruses.

## 2. Materials and Methods

### 2.1. Genetic Ecology of NDVs in Bangladesh

Complete coding sequences of the F gene and HN gene from NDV strains circulating in Bangladesh between 2010 and 2024 were retrieved from the NCBI GenBank database. But the limited number of complete HN dataset in Bangladesh was deemed insufficient for comparative analysis. Therefore, only the complete F gene sequences in Bangladesh were analyzed to determine prevalent genotypes, subgenotypes, host species, and annual distribution of isolates, based on the updated NDV classification system [26].

### 2.2. Evolutionary Relationships of Complete F Gene Sequences

Complete F gene sequences of Bangladeshi NDV isolates and reference strains of various genotypes/subgenotypes, along with their translated protein sequences, were retrieved from the NCBI GenBank database. Phylogenetic relationships were inferred using MEGA 11 software [31], supported by BLAST searches in GenBank. Prior to phylogenetic reconstruction, formal nucleotide substitution model testing was performed using the model selection function implemented in MEGA‐11. The optimal model was selected based on Bayesian information criterion (BIC) scores. Then, the phylogenetic tree was generated using the maximum likelihood method with the Poisson correction model [32], and statistical support was evaluated with 1000 bootstrap replicates [33]. Initial trees for heuristic searches were automatically generated using neighbor‐joining and BioNJ algorithms applied to pairwise distance matrices estimated by the Poisson model. The tree with the highest log‐likelihood value was selected. Evolutionary rate variation among sites was modeled with a discrete Gamma distribution (two categories, +G, with estimated parameters). The final tree was drawn to scale, with branch lengths representing the number of substitutions per site.

### 2.3. Evolutionary Divergence of F Gene Sequences

Evolutionary divergence among Bangladeshi NDV isolates within and between genotypes/subgenotypes was assessed using amino acid sequences of the complete F gene, applying the Poisson correction model [32]. Ambiguous positions were removed using the pairwise deletion option. All analyses were performed in MEGA 11 [31].

### 2.4. Amino Acid Variability in F Gene Sequences

Amino acid variability among Bangladeshi NDV isolates was examined by comparing complete F gene sequences across genotypes and subgenotypes. Variability in Bangladeshi isolates was also compared with commonly used NDV vaccine strains (LaSota, B1, and Mukteswar). The amino acid variability was conducted in MEGA 11 software [31], with multiple sequence alignments performed using the ClustalW algorithm [34].

### 2.5. Sequence Similarity Between Vaccine Strains and Field Isolates

Sequence similarity analyses were performed between the complete F gene sequences of vaccine strains (LaSota, B1, and Mukteswar) and circulating Bangladeshi NDV isolates across genotypes and subgenotypes. Vaccine strain sequences were retrieved from the NCBI GenBank database, and analyses were carried out in MEGA 11 [31] using ClustalW algorithm [34].

## 3. Results

### 3.1. Genetic Ecology of NDVs in Bangladesh

A total of 26 complete F gene sequences of NDV isolates from Bangladesh were analyzed, revealing genetically diverse strains circulating among multiple bird species. All isolates belonged to Class II and were classified into several genotypes and subgenotypes. Specifically, two isolates were assigned to genotype II, one to genotype VI (subgenotype VI.1.1, formerly VI.b), four to genotype VII (subgenotype VII.2), 16 to genotype XIII (11 in subgenotype XIII.2, four in XIII.2.3, and one unclassified), and three to genotype XXI (subgenotype XXI.1.2). Isolates from subgenotypes VI.1.1 and XXI.1.2 were recovered from pigeons, whereas isolates from genotypes II, VII.2, XIII.2, and XIII.2.3 originated from chickens.

All isolates were predicted to be virulent based on the presence of multibasic amino acid motifs such as “RRQKRF,” “RRKKRF,” or “RTKKRF” at the F protein cleavage site, except for genotype II isolates, which contained the monobasic motif “GRQGRL” characteristic of lentogenic strains. Notably, no complete F gene sequences of Bangladeshi NDV isolates were available in the NCBI GenBank database for the years 2011, 2013, 2014, 2015, 2019, and 2022 (Table 1).

**TABLE 1:** Complete F gene‐based NDV isolates found in Bangladesh during 2010–2024.

Sl no.	GenBank accession no.	Host	Isolation year	Cleavage site	Genotype/subgenotype
1	ON713864	Chicken	2018	GRQGRL	II
2	ON713865	Chicken	2018	GRQGRL	II
3	JX028552	Pigeon	2010	RRQKRF	VI.1.1
4	OP378144	Chicken	2020	RTKKRF	VII.2
5	OP378145	Chicken	2021	RRKKRF	VII.2
6	OP378146	Chicken	2021	RRKKRF	VII.2
7	OP378147	Chicken	2021	RTKKRF	VII.2
8	MK934286	Chicken	2010	RRQKRF	XIII.2
9	MK934287	Chicken	2010	RRQKRF	XIII.2
10	MK934288	Chicken	2010	RRQKRF	XIII.2
11	MK934289	Chicken	2010	RRQKRF	XIII.2
12	MK934290	Chicken	2010	RRQKRF	XIII.2
13	MK934291	Chicken	2012	RRQKRF	XIII.2
14	MK934292	Chicken	2012	RRQKRF	XIII.2
15	MK934293	Chicken	2012	RRQKRF	XIII.2
16	MK934294	Chicken	2016	RRQKRF	XIII.2
17	MK934295	Chicken	2017	RRQKRF	XIII.2
18	MK934296	Chicken	2017	RRQKRF	XIII.2
19	ON745442	Chicken	2018	RRQKRF	XIII
20	PQ333012	Chicken	2023	RRQKRF	XIII.2.3
21	PQ493420	Chicken	2024	RRQKRF	XIII.2.3
22	PQ657774	Chicken	2024	RRQKRF	XIII.2.3
23	PQ657775	Chicken	2024	RRQKRF	XIII.2.3
24	OP641847	Pigeon	2020	RRQKRF	XXI.1.2
25	OP641848	Pigeon	2020	RRQKRF	XXI.1.2
26	OP641849	Pigeon	2021	RRQKRF	XXI.1.2

### 3.2. Phylogenetic Analysis of Bangladeshi NDV Isolates

**FIGURE 1:**
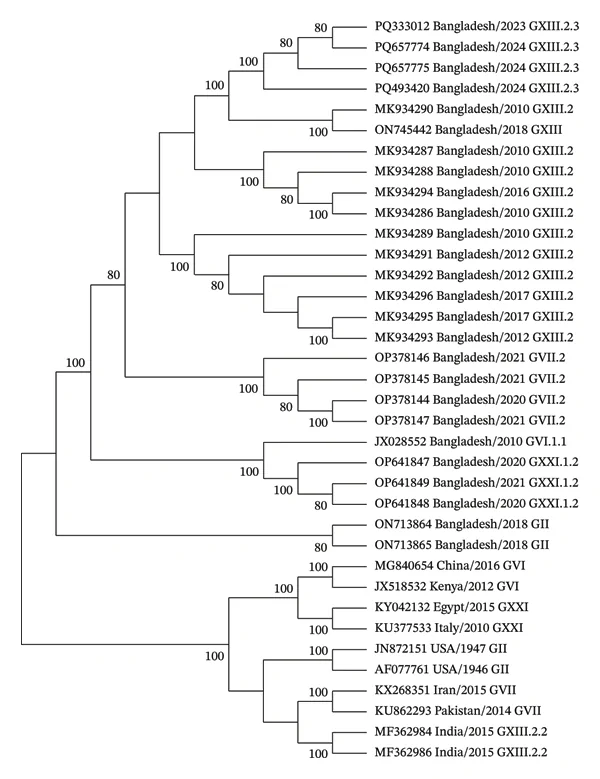
Phylogenetic tree of Bangladeshi NDV isolates, in which the evolutionary history was inferred by using the maximum likelihood method and Poisson correction model with 1000 bootstrap replicates. The tree with the highest log likelihood (−14336.31) is shown. The percentage of trees in which the associated taxa clustered together is shown next to the branches. Initial tree(s) for the heuristic search were obtained automatically by applying neighbor‐joining and BioNJ algorithms to a matrix of pairwise distances estimated using the Poisson model, and then selecting the topology with superior log likelihood value. A discrete gamma distribution was used to model evolutionary rate differences among sites (5 categories (+G, parameter = 0.8921)). This analysis involved 36 amino acid sequences. There were total of 1662 positions in the final dataset. Evolutionary analyses were conducted in MEGA11 software. Only bootstrap values above 70 are shown.

Phylogenetic analysis revealed close evolutionary relationships among isolates within genotype II, subgenotype VII.2, XIII.2.3, and XXI.1.2. By contrast, subgenotype XIII.2 isolates were distributed across separate subclades, indicating greater divergence within this group. Distinct clustering patterns were observed between genotypes and subgenotypes (Figure 1). BLAST analysis further showed that Bangladeshi isolates shared high sequence similarity (93.98%–99.96%) with isolates from India, Pakistan, Japan, Indonesia, and Jordan (Table 2).

**TABLE 2:** Evolutionary relationship of Bangladeshi NDV isolates with other countries’ isolates.

Isolates’ genotype/subgenotype	Similarity to isolates of other countries		
	Similarity (%)	Country	Host
II	99.93%–99.96%	India and Jordan	Chicken
VI.1.1	94.10	Pakistan	Pigeon
VII.2	98.43%–98.49%	India and Indonesia	Barn owls and eagles
XIII.2	93.98%–97%	India and Japan	Chicken
XIII.2.3	94.28%	Japan	Chicken
XXI.1.2	98%	Pakistan	Pigeon

### 3.3. Evolutionary Divergence of NDVs in Bangladesh

Analysis of 26 amino acid sequences of Bangladeshi NDV isolates, with a total alignment length of 553 positions, showed the following pairwise mean evolutionary distances: genotype II (0.02), subgenotype VII.2 (0.00–0.03), subgenotype XIII.2 (0.00–0.07), subgenotype XIII.2.3 (0.00–0.01), and subgenotype XXI.1.2 (0.00–0.02) (Supporting Tables 1.a–1.e). The overall mean evolutionary distances within each group were 0.02 (II), 0.02 (VII.2), 0.04 (XIII.2), 0.00 (XIII.2.3), and 0.02 (XXI.1.2).

### 3.4. Amino Acid Variability in F Gene Sequences of Bangladeshi NDV Isolates

Amino acid variability was examined across complete F gene sequences within each genotype and subgenotype. Genotype II isolates exhibited nine substitutions while retaining the conserved lentogenic cleavage site “GRQGRL.” Subgenotype VII.2 isolates, featuring “RRKKRF” and “RTKKRF” motifs, showed 14 variations. Subgenotype XIII.2 isolates carrying the virulent “RRQKRF” motif displayed 61 variable sites, whereas XIII.2.3 isolates showed only three substitutions. Subgenotype XXI.1.2 isolates, which also carried the “RRQKRF” motif, exhibited 13 variations.

**FIGURE 2:**
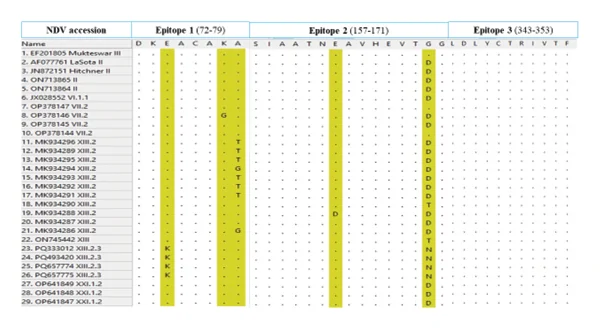
Amino acid variability at antigenic epitopes in F gene sequences of Bangladeshi NDVs compared to NDV vaccine strains used in Bangladesh in which antigenic variation in Epitope 1 and Epitope 2 were distinguished but Epitope 3 remained conserved.

**FIGURE 3:**
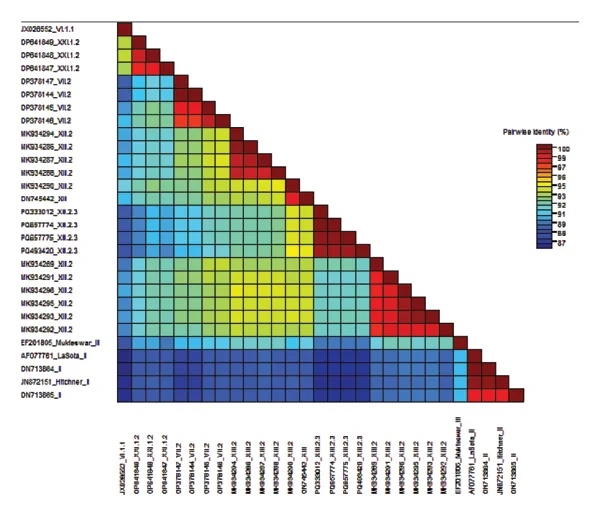
The pairwise identities‐based plot of F protein sequences aligned by MAFFT and displayed by sequence demarcation tool (SDT) software.

Notably, substitutions were detected in neutralizing Epitopes 1 and 2 of subgenotypes VII.2 and XIII.2, whereas Epitope 3 remained conserved across all isolates (Table 3). Further comparison with vaccine strains (LaSota, B1, and Mukteswar) confirmed substitutions at Epitope 1 in VII.2, XIII.2, and XIII.2.3. Compared with LaSota and B1, substitutions at Epitope 2 were observed in VII.2, XIII.2, and XIII.2.3; while compared with Mukteswar, substitutions at Epitope 2 were present in all circulating genotypes/subgenotypes in Bangladesh. Epitope 3 remained conserved across all isolates (Figure 2). Additionally, the sequence demarcation tool (SDT)–based analysis identified similarities across all strains isolated from the country (Figure 3). This highlights the genetic diversity and percussion this might have on the vaccine efficacies in the poultry sector.

**TABLE 3:** Amino acid variability in complete F gene sequences of circulating Bangladeshi NDVs within each genotype/subgenotype.

Isolates under genotype/subgenotype	Cleavage site	Variable amino acid position	Variables in neutralizing epitope of F protein
Genotype II	GRQGRL	17, 25, 264, 429, 441, 486, 520, 549, 550	Conserved
Subgenotype VII.2	RRKKRF	19, 24, 25, 31, 78, 97, 113, 170, 186, 190, 193, 235, 402, 442	Epitope 1 (K78G) and Epitope 2 (D170G)
Subgenotype XIII.2	RRQKRF	4, 5, 6, 8, 10–14, 18, 19, 24–26, 28, 29, 43, 49, 52, 66, 79, 97, 104, 107, 108, 110, 163, 170, 200, 208, 255, 270, 279, 286, 312, 321, 325, 328, 330, 338, 342, 364, 385, 395, 402, 442, 465, 479, 485, 486, 489, 492, 494, 496, 514, 516, 517, 520, 522, 530, 545	Epitope 1 (A79G/T) and Epitope 2 (E163D and D170T)
Subgenotype XIII.2.3	RRQKRF	4, 13, 63	Conserved
Subgenotype XXI.1.2	RRQKRF	16, 17, 19, 21, 150, 235, 294, 425, 470, 479, 487, 530, 537	Conserved

### 3.5. Sequence Similarity Between Vaccine Strains and Bangladeshi NDV Isolates

**FIGURE 4:**
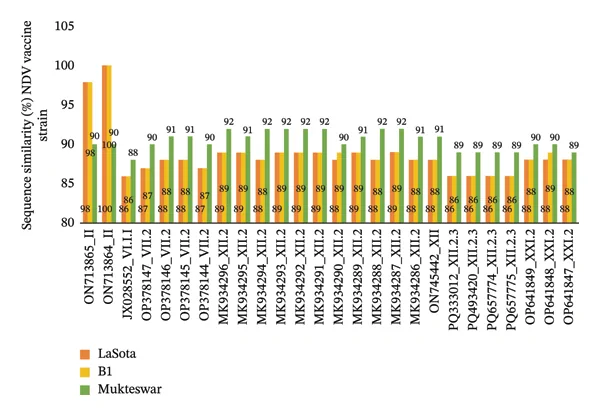
Sequence similarity (%) of NDV vaccine strains used in Bangladesh (LaSota, B1, and Mukteswar) compared to complete F gene sequences across different genotypes and subgenotypes of circulating Bangladeshi NDV isolates in which orange color indicates LaSota vaccine strain and its similarity (%) is labeled at inside base; gold color indicates B1 vaccine strain and its similarity (%) is labeled at the center; and green color indicates Mukteswar vaccine strain and its similarity (%) is labeled at outside end.

Comparative sequence similarity analysis revealed a considerable genetic mismatch between circulating NDV isolates and commonly used vaccine strains. LaSota and B1 showed high sequence similarity (98–100%) only with lentogenic strains of genotype II and moderate similarity (86%–89%) with virulent strains of genotypes VI.1.1, VII.2, XIII.2, XIII.2.3, and XXI.1.2. By contrast, Mukteswar exhibited 88%–92% sequence similarity with all circulating genotypes and subgenotypes in Bangladesh (Figure 4).

## 4. Discussion

NDV infects more than 200 avian species, although chickens remain the most susceptible host, followed by turkeys, pheasants, guinea fowl, partridges, quail, pigeons, and various wild birds that frequently serve as reservoirs [10, 35, 36]. In the present study, NDV was detected only in chickens and pigeons in Bangladesh. Pigeons are recognized as natural reservoirs of lentogenic and mesogenic NDV strains and contribute to viral maintenance and dissemination through mutation, genetic drift, recombination, and long‐distance movement, facilitating the emergence and spread of new genotypes [37].

Molecular characterization demonstrated that Class II NDVs currently circulating in Bangladesh comprise lentogenic genotype II together with virulent genotypes VI (VI.1.1), VII (VII.2), XIII (XIII.2 and XIII.2.3), and XXI (XXI.1.2) [26, 38, 39]. Phylogenetic and BLAST analyses further showed that Bangladeshi isolates shared close evolutionary relationships with strains reported from neighboring countries including India and Pakistan, as well as Jordan, Indonesia, and Japan, indicating frequent regional transmission and continuous viral evolution. Divergence analysis revealed limited diversity within most genotypes (overall pairwise distance 0.00–0.03), whereas genotype XIII.2 displayed comparatively greater heterogeneity (> 0.03), suggesting ongoing diversification of this lineage. These findings indicate that multiple virulent NDV genotypes are cocirculating in Bangladesh, creating continuous evolutionary pressure on currently used vaccines.

Despite the circulation of multiple virulent genotypes, vaccination programs in Bangladesh still rely predominantly on genotype II vaccines. LaSota (GenBank accession no. AF077761) and B1 (JN872151), both belonging to genotype II, are routinely administered to young chicks, whereas the mesogenic Mukteswar strain (EF201805), classified as genotype III, is commonly used in older birds [40, 41]. Although these vaccines effectively reduce mortality, they were developed decades ago from historical strains and therefore differ genetically from the currently circulating virulent genotypes VII and XIII that dominate outbreaks in Bangladesh.

The F protein is the principal protective antigen of NDV and one of the most important determinants of viral pathogenicity and antigenicity. The F protein cleavage site remains the most reliable molecular marker for virulence [42]. In this study, the canonical virulent motif RRQKRF predominated among Bangladeshi isolates, whereas alternative motifs RRKKRF and RTKKRF were identified within subgenotype VII.2. Previous studies have suggested that glutamine (*Q*) at the third position enhances membrane fusion efficiency and promotes cell‐to‐cell spread, thereby contributing to viral adaptation and evolution [43, 44].

More importantly, comparison of complete F protein sequences with the vaccine strains LaSota, B1 and Mukteswar demonstrated several amino acid substitutions within recognized neutralizing epitopes. Compared with genotype II vaccine strains (LaSota and B1), substitutions were detected at residues E74K, K78G, A79T/G (Epitope 1) and E163D, D170G, D170T, and D170N (Epitope 2). Relative to Mukteswar, additional substitutions including G170D, G170T, and related variants were observed among circulating genotypes II, VI.1.1, VII.2, XIII.2, XIII.2.3, and XXI.1.2. In contrast, Epitope 3 remained highly conserved across all Bangladeshi isolates.

The identified amino acid substitutions are biologically significant because they occur within major neutralizing epitopes of the F protein. Previous experimental studies have demonstrated that even single amino acid substitutions within these epitopes can alter antigenicity by modifying side‐chain polarity, charge distribution, steric configuration, or conformational flexibility, thereby reducing antibody binding affinity [45–50]. Furthermore, some substitutions may generate additional N‐linked glycosylation motifs capable of shielding antigenic epitopes from immune recognition. Consequently, mutations such as K78G, A79G/T, E163D, and D170G/T/N may alter both B‐cell and T‐cell epitope presentation, facilitating antigenic drift and reducing the neutralizing capacity of vaccine‐induced antibodies.

Consistent with these observations, sequence similarity analysis revealed that LaSota and B1 remained highly similar only to genotype II field isolates (98%–100%), whereas similarity declined substantially (approximately 80%–89%) against currently circulating virulent genotypes VI.1.1, VII.2, XIII.2, XIII.2.3 and XXI.1.2. Mukteswar exhibited only moderate similarity (88%–92%) with circulating viruses. Although genotype II vaccines generally continue to protect birds against mortality, increasing antigenic divergence between vaccine strains and circulating viruses likely contributes to incomplete protection, prolonged virus shedding and persistent field outbreaks [48, 51].

The present findings suggest that antigenic variation within the F protein is likely one of the major factors contributing to reduced vaccine effectiveness in Bangladesh. Nevertheless, immune escape cannot be attributed solely to amino acid substitutions because vaccine performance is also influenced by vaccination coverage, administration practices, maternal antibody interference, flock management, and farm biosecurity. In Bangladesh, weaknesses in biosecurity, mixed‐species farming, poor control of wild birds, incomplete flock vaccination, irregular booster schedules, and continued dependence on genotype II vaccines collectively create ecological and immunological pressures that favor viral persistence and evolution [52]. Therefore, F protein mutations probably act together with management‐related factors to facilitate vaccine breakthrough infections rather than causing complete immune escape independently.

Recent studies consistently demonstrate that genotype‐matched vaccines provide superior protection against contemporary NDV strains. Live and inactivated genotype VII vaccines, genotype XIII‐based vaccines, recombinant viral‐vectored vaccines, DNA vaccines, and thermostable genotype VII vaccines have all shown improved protection and significantly reduced virus shedding compared with conventional genotype II vaccines [53–61]. In Bangladesh, both genotype XIII.2‐ and genotype VII.2‐based inactivated vaccines provided almost complete protection against homologous challenge, whereas the commercial genotype II LaSota vaccine afforded only partial protection [41, 56]. These findings strongly support updating the national vaccination strategy toward genotype‐matched vaccines targeting the predominant VII and XIII lineages.

Future research should therefore prioritize continuous molecular surveillance of circulating NDVs together with detailed characterization of antigenic mutations within F protein to identify residues under positive selection. Such information will facilitate rational design of genotype‐specific or multivalent vaccines capable of providing broader cross‐protection against evolving Bangladeshi NDV strains while reducing virus shedding and limiting future outbreaks.

## 5. Conclusion

The genetic diversity of NDV genotypes and subgenotypes circulating in Bangladesh between 2010 and 2024 reveals the cocirculation of multiple strains, including novel and virulent variants. Subgenotype VII.2, associated with the fifth panzootic, and the recently detected a novel subgenotype XIII.2.3 highlight the dynamic evolutionary nature of NDV in the region and their potential implications for poultry health and production.

Although the present study did not include immune challenge or vaccine efficacy assays, previous studies have reported that genetic divergence between vaccine strains (commonly genotypes II and III) and circulating field strains may influence antigenic matching and potentially affect vaccine‐induced protection. Therefore, continuous molecular surveillance is essential to monitor evolving genotypes and to inform future vaccine design strategies.

Development of NDV vaccines particularly targeting predominant genotypes such as VII and XIII, along with optimized vaccination strategies, and strengthened biosecurity, may contribute to improved control of ND in Bangladesh.

## Author Contributions

Farah Zereen: methodology, data curation, software, formal analysis, and writing–original draft; Md. Abdur Rahman: methodology, data curation, software, formal analysis, and writing–original draft; Md. Golzar Hossain: writing–reviewing and editing and funding acquisition; Jahangir Alam, Masaru Shimada, Md. Tanvir Rahman, and Muhammad Munir: writing–reviewing and editing; Sukumar Saha: conceptualization, methodology, supervision, project administration, writing–reviewing and editing, and funding acquisition.

## Funding

This study was supported by Bangladesh Academy of Sciences, 2023/8/BAS.

## Conflicts of Interest

The authors declare no conflicts of interest.

## Supporting Information

Additional supporting information can be found online in the Supporting Information section.

## Supporting information


**Supporting Information** Divergence analysis showed low genetic diversity among the isolates of genotypes II, VII.2, XIII.2.3, and XXI.1.2, whereas genotype XIII.2 exhibited moderate heterogeneity. The higher overall mean distance (0.08) across all isolates indicates limited intergenotypic similarity and underscores the mutational potential of NDVs (Supporting Tables 1.a–1.e).

## Data Availability

Data sharing is not applicable to this article as no new data were created in this study.

## References

[1] Rima B. , Balkema-Buschmann A. , Dundon W. G. et al., ICTV Virus Taxonomy Profile: Paramyxoviridae, Journal of General Virology. (2019) 100, no. 12, 1593–1594, 10.1099/jgv.0.001328. 31609197 PMC7273325

[2] Mansour S. M. G. , ElBakrey R. M. , Mohamed F. F. et al., Avian Paramyxovirus Type 1 in Egypt: Epidemiology, Evolutionary Perspective, and Vaccine Approach, Frontiers in Veterinary Science. (2021) 8, 10.3389/fvets.2021.647462. PMC8320000 34336965

[3] Alexander D. J. , Newcastle Disease, British Poultry Science. (2001) 42, no. 1, 5–22, 10.1080/713655022. 11337967

[4] Alexander D. J. , Ecology and Epidemiology of Newcastle Disease, Avian Influenza and Newcastle Disease: A Field and Laboratory Manual. (2009) 19–26.

[5] Ballagi-Pordany A. , Wehmann E. , Herczeg J. , Belak S. , and Lomniczi B. , Identification and Grouping of Newcastle Disease Virus Strains by Restriction Site Analysis of a Region From the F Gene, Archives of Virology. (1996) 141, no. 2, 243–261, 10.1007/bf01718397. 8634018

[6] Chowdhury T. I. M. F. R. , Sarker A. J. , Amin M. M. , and Hossain W. I. M. A. , Studies of Newcastle Disease in Bangladesh, Bangladesh Veterinarian Journal. (1981) 15, 1–9.

[7] Rehman Z. U. , Ren S. , Butt S. L. et al., Newcastle Disease Virus Induced Pathologies Severely Affect the Exocrine and Endocrine Functions of the Pancreas in Chickens, Genes. (2021) 12, no. 4, 10.3390/genes12040495. PMC8067305 33805275

[8] Wajid A. , Maqsood Q. , Ben Said M. et al., Geographic Distribution and Genetic Diversity of Newcastle Disease Virus in Pigeons from Pakistan, Avian Pathology. (2024) 53, no. 2, 134–145, 10.1080/03079457.2023.2291107. 38037737

[9] Xiao S. , Nayak B. , Samuel A. , Paldurai A. , Kanabagattebasavarajappa M. , Prajitno T. Y. , Bharoto E. E. , Collins P. L. , and Samal S. K. , Generation by Reverse Genetics of an Effective, Stable, Live-Attenuated Newcastle Disease Virus Vaccine Based on a Currently Circulating, Highly Virulent Indonesian Strain, PLoS One. (2012) 7, no. 12, 10.1371/journal.pone.0052751. PMC3528709 23285174

[10] Jin J. , Zhao J. , Ren Y. , Zhong Q. , and Zhang G. , Contribution of HN Protein Length Diversity to Newcastle Disease Virus Virulence, Replication and Biological Activities, Scientific Reports. (2016) 6, no. 1, 10.1038/srep36890. PMC5105081 27833149

[11] Berihulay H. , Luo W. , Lao A. et al., Exploring the Genetic Basis of Newcastle Disease Virus in Chickens: A Comprehensive Review, Frontiers in Immunology. (2025) 16, 10.3389/fimmu.2025.1614794. PMC12245680 40655137

[12] Ma S. , Xia R. , Wu W. , and Duan Z. , Insights Into the Multifunctionality of Viral Glycoproteins F and HN in the Lifecycle and Pathogenesis of Newcastle Disease Virus: A Systematic Review, Veterinary Research. (2025) 56, no. 1, 10.1186/s13567-025-01647-0. PMC12590790 41194263

[13] Lu X. , Wang X. , Liu X. , and Liu X. , The Multifaceted Interactions Between Newcastle Disease Virus Proteins and Host Proteins: A Systematic Review, Virulence. (2024) 15, no. 1, 10.1080/21505594.2023.2299182. PMC10793697 38193514

[14] Snoeck C. J. , Owoade A. A. , Couacy-Hymann E. et al., High Genetic Diversity of Newcastle Disease Virus in Poultry in West and Central Africa: Cocirculation of Genotype XIV and Newly Defined Genotypes XVII and XVIII, Journal of Clinical Microbiology. (2013) 51, no. 7, 2250–2260, 10.1128/jcm.00684-13. 23658271 PMC3697698

[15] WOAH , Newcastle Disease (Infection With Newcastle Disease Virus), Manual of Diagnostic Tests and Vaccines for Terrestrial Animals: (Mammals, Birds and Bees), 2024, 1, 555–574.

[16] Ali A. A. H. , Abdallah F. M. , Farag G. K. , and Fatehi S. , An Overview on Virological Pathognesis, Clinical Manifestation, and Molecular Studies on Newcastle Disease Virus, European Journal of Molecular and Clinical Medicine. (2022) 9, no. 4, 2807–2824, https://ejmcm.com/pdf_19147_252376f6f087e9fbf06385528c6f4108.html.

[17] Fan W. , Wang Y. , Wang S. et al., Virulence in Newcastle Disease Virus: A Genotyping and Molecular Evolution Spectrum Perspective, Research in Veterinary Science. (2017) 111, 49–54, 10.1016/j.rvsc.2016.12.001. 27951521

[18] Moura V. M. B. D. , Susta L. , Cardenas-Garcia S. et al., Neuropathogenic Capacity of Lentogenic, Mesogenic, and Velogenic Newcastle Disease Virus Strains in Day-Old Chickens, Veterinary Pathology. (2016) 53, no. 1, 53–64, 10.1177/0300985815600504. 26395462

[19] Hamid H. , Campbell R. S. F. , and Lamichhane C. , The Pathology of Infection of Chickens With the Lentogenic V4 Strain of Newcastle Disease Virus, Avian Pathology. (1990) 19, no. 4, 687–696, 10.1080/03079459008418724. 18679982

[20] Walker P. J. , Siddell S. G. , Lefkowitz E. G. et al., Recent Changes to Virus Taxonomy Ratified by the International Committee on Taxonomy of Viruses, Archives of Virology. (2022) 167, no. 11, 2429–2440, 10.1007/s00705-022-05516-5. 35999326 PMC10088433

[21] Czeglédi A. , Ujvári D. , Somogyi E. , Wehmann E. , Werner O. , and Lomniczi B. , Third Genome Size Category of Avian Paramyxovirus Serotype 1 (Newcastle Disease Virus) and Evolutionary Implications, Virus Research. (2006) 120, no. 1-2, 36–48, 10.1016/j.virusres.2005.11.009. 16766077

[22] Kim L. M. , King D. J. , Suarez D. L. , Wong C. W. , and Afonso C. L. , Characterization of Class I Newcastle Disease Virus Isolates From Hong Kong Live Bird Markets and Detection Using Real-Time Reverse Transcription-PCR, Journal of Clinical Microbiology. (2007) 45, no. 4, 1310–1314, 10.1128/jcm.02594-06. 17287322 PMC1865838

[23] Kim L. M. , King D. J. , Curry P. E. et al., Phylogenetic Diversity Among Low-Virulence Newcastle Disease Viruses From Waterfowl and Shorebirds and Comparison of Genotype Distributions to Those of Poultry-Origin Isolates, Journal of Virology. (2007) 81, no. 22, 12641–12653, 10.1128/jvi.00843-07. 17855536 PMC2169019

[24] Welch C. N. , Shittu I. , Abolnik C. et al., Genomic Comparison of Newcastle Disease Viruses Isolated in Nigeria Between 2002 and 2015 Reveals Circulation of Highly Diverse Genotypes and Spillover Into Wild Birds, Archives of Virology. (2019) 164, no. 8, 2031–2047, 10.1007/s00705-019-04288-9. 31123963

[25] Suarez D. L. , Miller P. J. , Koch G. , Mundt E. , and Rautenschlein S. , Swayne D. E. , Newcastle Disease, Other Avian Paramyxoviruses, and Avian metapneumovirus Infections, Diseases of Poultry, 2020, 14th edition, Wiley-Blackwell, Hoboken, NJ, USA, 111–166.

[26] Dimitrov K. M. , Abolnik C. , Afonso C. L. et al., Updated Unified Phylogenetic Classification System and Revised Nomenclature for Newcastle Disease Virus, Infection, Genetics and Evolution. (2019) 74, 10.1016/j.meegid.2019.103917. PMC6876278 31200111

[27] Nath B. and Kumar S. , Emerging Variant of Genotype XIII Newcastle Disease Virus From Northeast India, Acta Tropica. (August 2017) 172, 64–69, 10.1016/j.actatropica.2017.04.018. 28450210

[28] Panda A. , Huang Z. , Elankumaran S. , Rockemann D. D. , and Samal S. K. , Role of Fusion Protein Cleavage Site in the Virulence of Newcastle Disease Virus, Microbial Pathogenesis. (2004) 36, no. 1, 1–10, 10.1016/j.micpath.2003.07.003. 14643634 PMC7125746

[29] Puro K. and Sen A. , Newcastle Disease in Backyard Poultry Rearing in the Northeastern States of India: Challenges and Control Strategies, Frontiers in Veterinary Science. (2022) 9, 10.3389/fvets.2022.799813. PMC9021565 35464373

[30] Zeb M. T. , Dumont E. , Khan M. T. , Shehzadi A. , and Ahmad I. , Multi-Epitopic Peptide Vaccine Against Newcastle Disease Virus: Molecular Dynamics Simulation and Experimental Validation, Vaccines. (2024) 12, no. 11, 10.3390/vaccines12111250. PMC11598688 39591153

[31] Tamura K. , Stecher G. , and Kumar S. , MEGA 11: Molecular Evolutionary Genetics Analysis Version 11, Molecular Biology and Evolution. (2021) 38, no. 7, 3022–3027, 10.1093/molbev/msab120. 33892491 PMC8233496

[32] Zuckerkandl E. and Pauling L. , Bryson V. and Vogel H. J. , Evolutionary Divergence and Convergence in Proteins, Evolving Genes and Proteins, 1965, Academic Press, New York, 97–166.

[33] Felsenstein J. , Confidence Limits on Phylogenies: An Approach Using the Bootstrap, Evolution. (1985) 39, no. 4, 783–791, 10.1111/j.1558-5646.1985.tb00420.x. 28561359

[34] Thompson J. D. , Higgins D. G. , and Gibson T. J. , CLUSTAL W: Improving the Sensitivity of Progressive Multiple Sequence Alignment Through Sequence Weighting, Position-Specific Gap Penalties and Weight Matrix Choice, Nucleic Acids Research. (1994) 22, no. 22, 4673–4680, 10.1093/nar/22.22.4673. 7984417 PMC308517

[35] Getabalew M. , Alemneh T. , Akeberegn D. , Getahun D. , and Zewdie D. , Epidemiology, Diagnosis & Prevention of Newcastle Disease in Poultry, American Journal of Biomedical Science and Research. (2019) 3, no. 1, 50–59, 10.34297/AJBSR.2019.03.000632.

[36] Snoeck C. J. , Adeyanju A. T. , Owoade A. A. et al., Genetic Diversity of Newcastle Disease Virus in Wild Birds and Pigeons in West Africa, Applied and Environmental Microbiology. (2013) 79, no. 24, 7867–7874, 10.1128/aem.02716-13. 24123735 PMC3837833

[37] Ellakany H. F. , Elbestawy A. R. , Abd El-Hamid H. S. et al., Role of Pigeons in the Transmission of Avian Avulavirus (Newcastle Disease-Genotype VIId) to Chickens, Animals. (2019) 9, no. 6, 10.3390/ani9060338. PMC6617408 31185682

[38] Kochiganti S. , Rajkhowa T. K. , Kiran J. , Zodinpui D. , and Choudhury F. A. , Molecular Characterization of Class II Newcastle Disease Virus From Field Outbreaks in Mizoram, India, Indian Journal of Animal Research. (2024) 10.18805/IJAR.B-5241.

[39] Zereen F. , Rahman M. A. , Hossain M. G. , Alam J. , Shimada M. , and Saha S. , First Report of the Emergence of Novel Sub-Genotype XIII.2.3 of Newcastle Disease Virus in Chickens From Selected Regions of Bangladesh, Infection, Genetics and Evolution. (2024) 130, 10.1016/j.meegid.2025.105742. 40120636

[40] Barman L. R. , Islam M. N. , Flensburg M. F. , Permin A. , Petersen S. L. , and Islam M. R. , Newcastle Disease Vaccination Regimen Comprising Both Lentogenic and Mesogenic Strains is More Effective than Lentogenic Strain Only, Bangladesh Veterinarian. (2010) 27, no. 1, 1–7, 10.3329/bvet.v27i1.5908.

[41] Hossain I. , Subarna J. F. , Kabiraj C. K. et al., A Booster With a Genotype-Matched Inactivated Newcastle Disease Virus (NDV) Vaccine Candidate Provides Better Protection Against a Virulent Genotype XIII.2 Virus, Vaccines. (2023) 11, no. 5, 10.3390/vaccines11051005. PMC10221827 37243108

[42] Wisemana A. , Shabbata M. , Cohena A. et al., Point Mutation Revealed the Resurgence of Sub-Genotype VII.2 Newcastle Disease Virus in Israel, Avian Pathology. (2022) 51, no. 3, 236–243, 10.1080/03079457.2022.2040730. 35234543

[43] Samal S. , Khattar S. K. , Paldurai A. et al., Mutations in the Cytoplasmic Domain of the Newcastle Disease Virus Fusion Protein Confer Hyperfusogenic Phenotypes Modulating Viral Replication and Pathogenicity, Journal of Virology. (2013) 87, no. 18, 10083–10093, 10.1128/jvi.01446-13. 23843643 PMC3754023

[44] Wang Y. , Yu W. , Huo N. et al., Comprehensive Analysis of Amino Acid Sequence Diversity at the F Protein Cleavage Site of Newcastle Disease Virus in Fusogenic Activity, PLoS One. (2017) 12, no. 9, 10.1371/journal.pone.0183923. PMC5580908 28863165

[45] Toyoda T. , Gotoh B. , Sakaguchi T. , Kida H. , and Nagai Y. , Identification of Amino Acids Relevant to Three Antigenic Determinants on the Fusion Protein of Newcastle Disease Virus That are Involved in Fusion Inhibition and Neutralization, Journal of Virology. (1998) 62, no. 11, 4427–4430, 10.1128/jvi.62.11.4427-4430.1988. PMC253888 2459417

[46] Yusoff K. , Nesbit M. , McCartney H. et al., Location of Neutralizing Epitopes on the Fusion Protein of Newcastle Disease Virus Strain Beaudette C, Journal of General Virology. (1989) 70, no. 11, 3105–3109, 10.1099/0022-1317-70-11-3105. 2479718

[47] Neyt C. , Geliebter J. , Slaoui M. , Morales D. , Meulemans G. , and Burny A. , Mutations Located on Both Fl and F2 Subunits of the Newcastle Disease Virus Fusion Protein Confer Resistance to Neutralization With Monoclonal Antibodies, Journal of Virology. (1989) 63, no. 2, 952–954, 10.1128/jvi.63.2.952-954.1989. 2463386 PMC247772

[48] Omony J. B. , Wanyana A. , Mugimba K. K. et al., Epitope Peptide-Based Predication and Other Functional Regions of Antigenic F and HN Proteins of Waterfowl and Poultry Avian Avulavirus Serotype-1 Isolates From Uganda, Frontiers in Veterinary Science. (2021) 8, 10.3389/fvets.2021.610375. PMC8240872 34212016

[49] Shalit Y. and Tuvi-Arad I. , Side Chain Flexibility and the Symmetry of Protein Homodimers, PLoS One. (2020) 15, no. 7, 10.1371/journal.pone.0235863. PMC7380632 32706779

[50] Karim M. S. , Selim A. , Arafa A. , Hussein H. A. , and Ahmed A. E. , Molecular Characterization of Full Fusion Protein (F) of Newcastle Disease Virus Genotype VIId Isolated From Egypt During 2012–2016, Veterinary World. (2018) 11, no. 7, 930–938, 10.14202/vetworld.2018.930-938. 30147262 PMC6097568

[51] Saha S. M. I. , Rahman M. M. , and Alam K. M. T. , Efficacy of an Inactivated Newcastle Disease Virus Vaccine Prepared From a Local Isolate, Bangladesh Veterinary Journal. (1998) 32, 57–62.

[52] Fatima A. , Spaul M. R. , Khan H. et al., Unveiling the Hidden Link of Biosecurity in Preventing Vaccine Failure in Livestock: An Updated Review, Biomedical Communications. (2025) 1, no. 1, 18–29, 10.71320/bcs.0003.

[53] Wang X. , Yao Y. , Yang W. et al., Development and Evaluation of a Novel Chimeric Genotype VII Newcastle Disease Vaccine: Overcoming Maternal Antibody Interference and Spray Administration, Veterinary Sciences. (2024) 11, 10.3390/vetsci11110532. PMC11599132 39591306

[54] Amoia C. F. , Chengula A. A. , Hakizimana J. N. , Wambura P. N. , Munir M. , Misinzo G. , and Weger-Lucarell J. , Development of a Genotype-Matched Newcastle Disease DNA Vaccine Candidate Adjuvanted With IL-28b for the Control of Targeted Velogenic Strains of Newcastle Disease Virus in Africa, Veterinary Research Communications. (2024) 49, no. 1, 10.1007/s11259-024-10590-y. PMC11588948 39585481

[55] Abdallah Mouhamed A. , Lee J. , Kim D.-H. , and Song C.-S. , Comparative Protective Efficacy of a Newly Generated Live Recombinant Thermostable Highly Attenuated Vaccine rK148/GVII-F Using a Single Regimen Against Lethal NDV GVII.1.1, Avian Pathology. (2024) 53, no. Issue 1, 14–32, 10.1080/03079457.2023.2263395. 38009206

[56] Haque M. A. , Haque M. E. , Parvin M. K. et al., Determination of Immunogenicity of an Inactivated ND-Vaccine Developed Experimentally With Newcastle Disease Virus (Genotype VII.2) Local Isolates of Bangladesh, Frontiers in Immunology. (2024) 15, 10.3389/fimmu.2024.1482314. PMC11576377 39569197

[57] Boravleva E. , Treshchalina A. , Gordeeva D. et al., Genotype I Newcastle Disease Virus, Isolated From Wild Duck, Can Protect Chickens Against Newcastle Disease Caused by Genotype VII, Pathogens. (2025) 14, no. 4, 10.3390/pathogens14040380. PMC12030387 40333124

[58] Hou C. , Ni R. , Zhao L. et al., A Novel Chimpanzee Adenovirus Vector Vaccine for Protection Against Infectious Bronchitis and Newcastle Disease in Chickens, Veterinary Research. (2025) 56, no. 1, 10.1186/s13567-025-01528-6. PMC12083102 40375108

[59] Hassanzadeh M. , Abedi M. , Bashashati M. , Yousefi A. R. , Abdoshah M. , and Mirzai S. , Evaluation of the Newcastle Disease Virus Genotype VII–Mismatched Vaccines in SPF Chickens: A Challenge Efficacy Study, Veterinary and Animal Science. (2024) 24, 10.1016/j.vas.2024.100348. PMC11016800 38623086

[60] Li Y. , Rehman Z. U. , Li M. et al., Comparison of the Protective Antigen Variabilities of Prevalent Newcastle Disease Viruses in Response to Homologous/Heterologous Genotype Vaccines, Poultry Science. (2021) 100, no. 8, 10.1016/j.psj.2021.101267. PMC8267594 34237546

[61] Ananda Kumar B. S. , Panickan S. , Bindu S. et al., Immunogenicity and Protective Efficacy of an Inactivated Newcastle Disease Virus Vaccine Encapsulated in Poly-(Lactic-Co-Glycolic Acid) Nanoparticles, Poultry Science. (2023) 102, no. 6, 10.1016/j.psj.2023.102679. PMC10160591 37116285

