# Supplementary material for: Molecular Characterization of Publicly Available NDV F Gene Sequences From Bangladesh (2010–2024)
Source: Vet Med Int. 2026 Aug 24;2026:8821675. doi: 10.1155/vmi/8821675 (PMC13501252; doi:10.1155/vmi/8821675)
Supplement: Supplementary file 1 [file VMI-2026-8821675-s001.docx]

***Supplementary Table 1.a Evolutionary Divergence between genotype II isolates***

| II | ON713865 | ON713864 |
| --- | --- | --- |
| ON713865 |  |  |
| ON713864 | 0.02 |  |

***Supplementary table 1.b Evolutionary Divergence among sub-genotype VII.2 isolates***

| VII.2 | OP378147 | OP378146 | OP378145 | OP378144 |
| --- | --- | --- | --- | --- |
| OP378147 |  |  |  |  |
| OP378146 | 0.03 |  |  |  |
| OP378145 | 0.02 | 0.01 |  |  |
| OP378144 | 0.00 | 0.03 | 0.02 |  |

***Supplementary table 1.c Evolutionary Divergence among sub-genotype XIII.2 isolates***

| XIII.2 | MK934296 | MK934289 | MK934295 | MK934294 | MK934293 | MK934292 | MK934291 | MK934290 | MK934288 | MK934287 | MK934286 |
| --- | --- | --- | --- | --- | --- | --- | --- | --- | --- | --- | --- |
| MK934296 |  |  |  |  |  |  |  |  |  |  |  |
| MK934289 | 0.02 |  |  |  |  |  |  |  |  |  |  |
| MK934295 | 0.01 | 0.02 |  |  |  |  |  |  |  |  |  |
| MK934294 | 0.06 | 0.07 | 0.06 |  |  |  |  |  |  |  |  |
| MK934293 | 0.00 | 0.02 | 0.00 | 0.06 |  |  |  |  |  |  |  |
| MK934292 | 0.01 | 0.02 | 0.01 | 0.07 | 0.01 |  |  |  |  |  |  |
| MK934291 | 0.01 | 0.02 | 0.01 | 0.06 | 0.01 | 0.01 |  |  |  |  |  |
| MK934290 | 0.06 | 0.07 | 0.06 | 0.06 | 0.06 | 0.07 | 0.07 |  |  |  |  |
| MK934288 | 0.06 | 0.07 | 0.06 | 0.01 | 0.06 | 0.07 | 0.06 | 0.06 |  |  |  |
| MK934287 | 0.06 | 0.07 | 0.06 | 0.01 | 0.06 | 0.07 | 0.06 | 0.06 | 0.01 |  |  |
| MK934286 | 0.06 | 0.07 | 0.06 | 0.00 | 0.06 | 0.07 | 0.06 | 0.06 | 0.01 | 0.01 |  |

***Supplementary table 1.d Evolutionary Divergence among sub-genotype XIII.2.3 isolates***

| XIII.2.3 | PQ333012 | PQ493420 | PQ657774 | PQ657775 |
| --- | --- | --- | --- | --- |
| PQ333012 |  |  |  |  |
| PQ493420 | 0.00 |  |  |  |
| PQ657774 | 0.00 | 0.00 |  |  |
| PQ657775 | 0.00 | 0.01 | 0.00 |  |

***Supplementary table 1.e Evolutionary Divergence among sub-genotype XXI.1.2 isolates***

| XXI.1.2 | OP641849 | OP641848 | OP641847 |
| --- | --- | --- | --- |
| OP641849 |  |  |  |
| OP641848 | 0.01 |  |  |
| OP641847 | 0.02 | 0.02 |  |
